# Preparation of Aluminum-Based Superhydrophobic Surfaces for Fog Collection by Bioinspired Sarracenia Microstructures

**DOI:** 10.3390/biomimetics9090535

**Published:** 2024-09-04

**Authors:** Yunjie Guo, Jie Li, Lisheng Ma, Wentian Shi, Yuke Wang, Shuo Fu, Yanning Lu

**Affiliations:** 1 School of Computer and Artificial Intelligence, Beijing Technology and Business University, Beijing 100048, China; guovt@sina.com (Y.G.); shiwt@th.btbu.edu.cn (W.S.); wyk19991101@163.com (Y.W.); 13522434209@163.com (S.F.); m15976500101@163.com (Y.L.); 2 College of Marine and Electrical Engineering, Jiangsu Maritime Institute, Nanjing 211106, China; mls@jmi.edu.cn

**Keywords:** bioinspired, superhydrophobic, hierarchical structure, fog collection

## Abstract

Freshwater shortage is a growing problem. Inspired by the Sarracenia trichome fog-trapping and ultrafast water-transport structure, a series of hierarchical textured surfaces with high-low ribs with different wettabilities was prepared based on laser processing combined with dip modification. Through fog-collection performance tests, it was found that the samples with superhydrophobicity and low adhesion had the best fog-collection effect. In addition, it was observed that the fog-collection process of different microstructured samples was significantly different, and it was analysed that the fog-collection process was composed of two aspects: directional condensation and directional transport of droplets, which were affected by the low ribs number and rib height ratio. A design parameter was given to create the Sarracenia trichome-like structure to achieve a fast water transport mode. This study provides a good reference for the development and preparation of fog-collection surfaces.

## 1. Introduction

Water scarcity is a problem that cannot be ignored and urgently needs to be solved in certain environmentally hostile areas. In recent years, surfaces with water-collecting functions have attracted the attention of many researchers [1,2,3,4]. Through observation and contemplation, researchers have been inspired by the fog-collecting behaviors of plants and animals in nature and have designed and prepared multifunctional bionic surfaces with water-collecting properties based on bionic science [5,6,7,8,9,10]. For example, cactus has a conical multistage structure of needle-like burrs, which not only reduces water loss, but also realizes the spontaneous movement of triggered water droplets and adapts it to the desert environment [11,12,13]. Inspired by it, Feng et al. [14] fabricated a spontaneously fog-harvest system (NAIPS) based on the slippery-superhydrophilic-patterned surface. The system can present a good water-collection effect, with a collection efficiency of up to 2166 ± 71 mg cm^−2^ h^−1^, which is increased by 139% compared to that of a homogeneous slippery surface. Spider silk is able to collect water in humid air and transport it directionally, thanks to its multi-scale and periodic structure formed by alternating rows of spindle knots and slender joints [15]. Inspired by spider silk, Huan et al. [16] obtained a novel bioinspired hydrophilic dual-thread spider silk fiber (HDSSF) via a simple dipping coating method. Compared to normal bioinspired spider silk fibers, a totally different fog-harvesting mode was observed. It allows rapid transport and directional collection of droplets, resulting in a 590% increase in fog-harvesting efficiency.

In recent years, Chen et al. [17] reported that Sarracenia trichome, a plant with hierarchical microscale and nanoscale structures on its surface, can achieve ultrafast directional water transport, attributed to the unique hierarchical microchannel organization of the trichomes, which is up to three orders of magnitude faster than that in cactus spine and spider silk. Xu successfully prepared a hierarchical groove surface for low-temperature fog collection by WEDM. It assists in droplet freeze prevention and water transport at low temperatures after fluorination and oil injection treatments, which improves fog-collection performance [18]. Inspired by Sarracenia trichome and cactus spine, Wang et al. designed and fabricated superhydrophilic surfaces with multiple bionic combinations of hierarchical structure, which exhibited excellent self-propelled water transport and efficient fog collection compared to single bionic structure and non-hierarchical structure [19].

Surface wettability is mainly affected by the morphology of the structure and surface energy. The chemical composition of the surface determines whether the surface is hydrophobic or hydrophilic, and its structure usually determines the degree of hydrophobicity or hydrophilicity. Therefore, the surface structure is likely to be one of the factors affecting the efficiency of fog collection [20,21,22]. In this study, a series of different hierarchical textured superhydrophobic surfaces on aluminum alloys, inspired by the trichome structure of Sarracenia, were fabricated and investigated for their fog-collection behavior. Different microstructures and nanostructures, wettability, and adhesion properties were obtained by laser processing and dip modification, and the required 18 sample surfaces were prepared to explore the effect of these differences on the fog-collection behavior. It is worth noting that in this study, the superhydrophobic surfaces was used for fog collection instead of the superhydrophilic surfaces. This is because the prepared superhydrophilic samples are easily oxidized in air and become hydrophobic, which affects the fog-collection results. In addition, due to the superhydrophilicity of the surface during the fog-collection process, its overall appearance of a thin film of water, with no obvious difference between samples of different bionic microstructures, does not facilitate further observation and analysis.

Compared with single bionic structure, the integrated structure has better water-transport effect, but there are fewer studies on the structural influence of Sarracenia trichomes on water transport and fog collection alone. We know that the hierarchical microstructures on the surface of Sarracenia are able to form a water film when wetted, which confers ultrafast water transport property, and thus its structure is an important factor affecting water transport. This study allows researchers to think about the factors affecting fog-collection behavior and opens up more possibilities for microfluidic and biomedicine.

## 2. Experimental Section

### 2.1. Materials

A total of 7075 aluminum alloys were used as the experimental materials and were purchased from Guangdong Jingui Metal Materials Co., Ltd. (Dongguan, China) sample size is 20 mm × 20 mm × 3 mm. Absolute ethanol (≥97%), N-hexane (≥98%), 1H, 1H, 2H, 2H-perfluorodecyltri-ethoxysilane (C_16_F_17_H_19_O_3_Si, FAS) (≥97%) were purchased from Beijing InnoChem Science & Technology Co., Ltd. (Bejing, China). Deionized water was made in the laboratory. All chemical reagents are of analytical grade.

### 2.2. Preparation of Surface Structures

**Figure 1:**
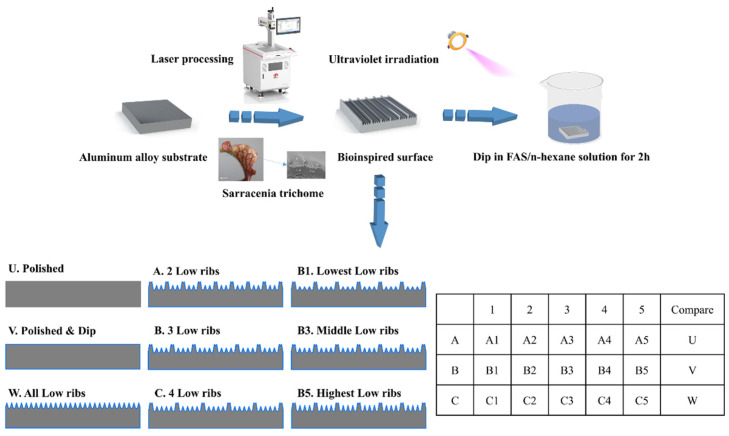
The schematic diagram of the experimental procedure.

Aluminum alloy samples were sanded and polished, ultrasonically cleaned with absolute ethanol and deionized water, and blown dry with N_2_ gas. The samples were prepared as follows: (1) Using a fiber laser-marking machine (LSF20, Wuhan Huaguang Laser Engineering Co., Ltd., Wuhan, China), in which the laser operates at a wavelength of 1064 nm, the focal spot size is 0.06 mm, and a series of straight lines are processed on the surface with an output power of 16 W. The number of low ribs of the samples from A to C is gradually increasing, and the rib height ratio (the ratio of the height of high ribs to that of low ribs) of samples from 1 to 5 is gradually decreasing, as shown in Figure 1. (2) Samples U, V, and W were used as control groups, where U and V were not laser processed and W was a full low-rib sample. The laser scanning speed was 500 mm/s, the frequency was 20 kHz, and the surface processing area was the same for all samples (error ≤ 0.3%). (3) The samples, except sample U, were exposed to UV light for 1 h, then immersed in a 1.5% (*v*/*v*) n-hexane solution containing FAS for 2 h, and finally dried in an oven at 90° for 1 h. A schematic diagram of the experimental procedure is shown in Figure 1.

### 2.3. Characterization

Microscopic morphology and composition of samples were characterized by scanning electron microscope (SEM) (Phenom XL, Phenom Scientific, Eindhoven, the Netherlands). An automatic stereo zoom microscope (SZ-2000, ASI, Eugene, OR, USA) was used to obtain the three-dimensional topographic map of the surface and contour curve of the profile. The surface wettability was evaluated with a contact angle tester (XG-CAM, XYCXIE, Shanghai, China) by measuring its static contact angle of 2 μL deionized water at five different positions. A commercial humidifier (Beiwei, 7.8 LX, rated fog output: 0.45 Lh^−1^, outlet inner diameter: 17.8 mm) (Shenzhen, China). was used, the sample was fixed vertically on the holder with an adhesive, and the distance between the humidifier and the sample was about 8 cm, so that the efficiency of the fog collection on the surface of the sample was observed and tested in the closed indoor ambient conditions (Temperature = 24.5 ± 1 °C, Relative humidity = 34.5 ± 3%).

## 3. Results and Discussion

### 3.1. Morphology

**Figure 2:**
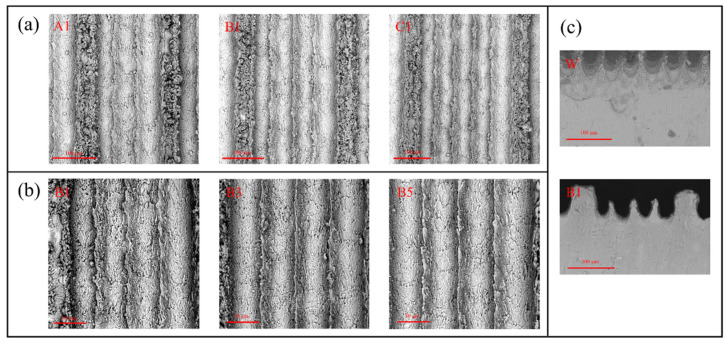
SEM images of the sample surface. (**a**) Top view of samples A1, B1, and C1. (**b**) Top view of samples B1, B3, and B5. (**c**) Front view of samples W and B1.

The microscopic morphology of the samples is shown in Figure 2, where Figure 2a shows the top view of the surfaces of samples with different low rib numbers, Figure 2b shows the top view of the surfaces of samples with different rib height ratio, and Figure 2c shows the front view of the surfaces of sample W and sample B1. Both high and low ribs are formed by sputtering on the surface of the laser-processed aluminum alloy. It was found that the height of the ribs is affected by adjusting the spacing of the laser lines in a certain pattern. For example, under the above laser parameters and processing times, the spacing of the lines is lower than 50 µm, which does not result in the formation of a neat and clear rib structure. The spacing of the lines is increased from 50 µm to 70 µm, the ribs width are almost the same, their height are increased gradually, and the height of the ribs formed by sputtering reaches the maximum value when the spacing of the lines is 70 µm. As the line spacing increases from 70 µm to 120 µm, the ribs width gradually increases and the rib height gradually decreases, with the highest level becoming more and more flat. With a line spacing greater than 120 µm, no rib structure can be formed, and only a slight sputtering effect can be observed. As shown in Figure 2b, the ribs are uniformly distributed, with uniform height and width, and micrometer scale pits and protrusions appear on the surface of the ribs, which are formed by localized melting and vaporization during the sputtering process. Compared with the high ribs, the surface particles and protrusion structures of the low ribs are finer, denser, and have smaller pore sizes than those of the high ribs. UV irradiation was able to promote the hydrolyzing process of FAS in n-hexane and the formation of in situ hydroxylation on the surface to achieve chemisorption. So, dip treatment covers the surface with a dense FAS molecular film, so this is a composite hierarchical microstructure with a more hydrophobic surface.

**Figure 3:**
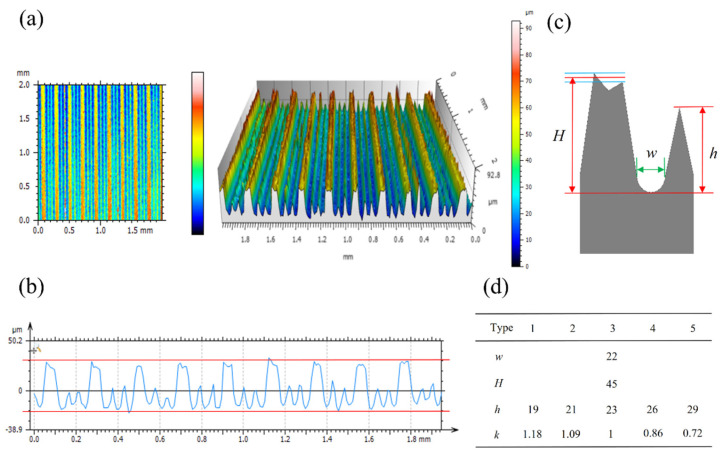
3D morphology of the sample surface. (**a**) Extraction area and 3D morphology. (**b**) Contour curve of the extracted profiles. (**c**) Structural parameters. (**d**) Coefficient of the rib height difference in the microchannel. Where *H* is High rib height, *h* is Low rib height, and *w* is Microchannel width.

Figure 3a shows the scanned area of 2 mm × 2 mm extracted from the surface of sample A3 and the 3D morphology view, and Figure 3b shows the contour curve of the extracted profile in this area. From the figure, it can be seen that the height difference between the high ribs and the low ribs is very obvious, and the surface structure has good integrity. The contour curves show that the shape and size of the high ribs and low ribs are fairly consistent, and the statistics of the structural parameters were carried out by extracting 10 regions of the same sample and 10 profiles from each region. Due to the uneven height of the protrusions at the top of the high ribs, the main structural parameters (High rib height *H*, Low rib height *h*, and Microchannel width *w*) were defined as shown in Figure 3c. Since the line spacing of the high ribs is the same for all the samples, the high ribs’ height should be the same, which is the same as the statistical result, and the obtained high ribs’ height is 45 µm. In Figure 3b, the difference between the highest and the lowest part of the surface structure is 50 µm, which indicates that the error of the data is very low. Based on the obtained structural parameters, the coefficient of the rib height difference in the microchannel k=(H−h)/w, was calculated as shown in Figure 3d.

### 3.2. Wettability and Durability

**Figure 4:**
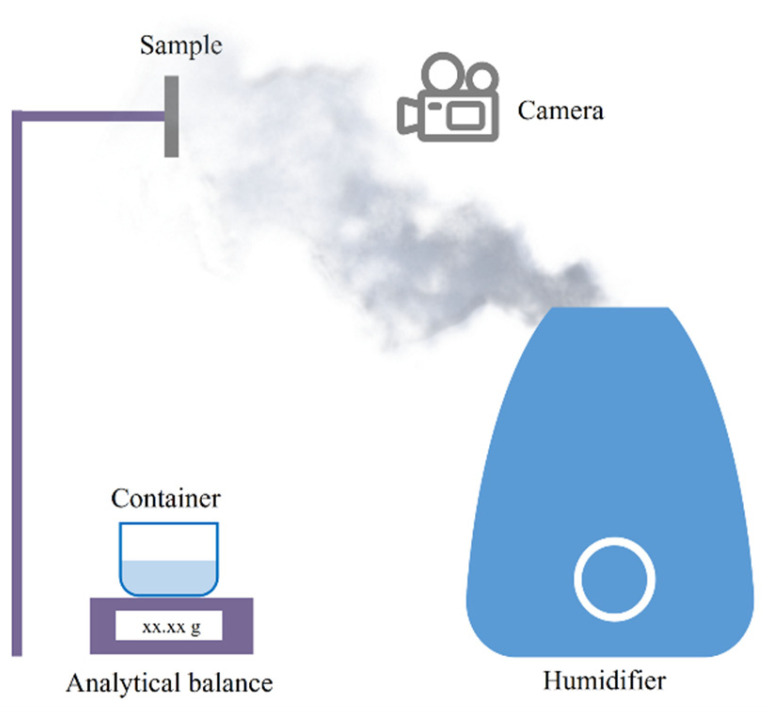
The schematic diagram of the fog-collection test device.

Figure 4 is a schematic diagram of the experimental device for the fog-collection test, where the sample is fixed vertically on a holder and the condensed droplets slide vertically down into the container.

**Figure 5:**
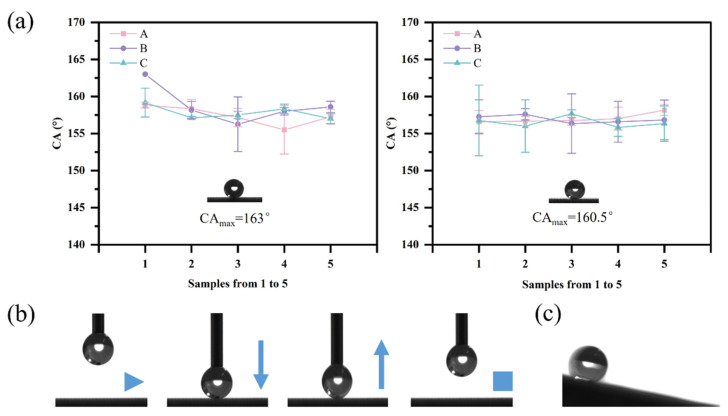
Wettability of sample surface. (**a**) Static contact angle. Samples from A to C with different number of low ribs from 2 to 4. (**b**) Low adhesion. (**c**) Rolling on the inclined surface.

The wettability of the surface is usually influenced by both low surface energy and rough microstructure. The surface energy was reduced by designing Sarracenia bionic microstructures and the fluoridation treatment to make the surface have good hydrophobicity, under the same fluoridation treatment, aiming to investigate the effect of the low rib number and rib height ratio on the hydrophobicity of the surface from the perspective of the bionic structure. The static contact angles of all samples before and after fog collection were measured separately, as shown in Figure 5a. The difference in contact angle of the samples before and after fog collection showed little overall change, with the contact angle of the control sample U being 76° and the contact angle of sample W decreasing from 158.41° to 154.67°. It is worth noting that the contact angle of sample V decreased from 117.75° to 86°, changing from hydrophobicity to hydrophilicity, which indicates that the fog collection has a great effect on the smooth surfaces after fluoridation treatment. This is because compared with the smooth surface, the surfaces of the samples with the bionic Sarracenia structure all reach superhydrophobicity, the contact area of the droplets with the surface is smaller, and it is featured with anisotropic wettability, so the condensation volume of the droplets is much less affected by the rib constraints than that of the smooth surfaces, which reduces the damage of the collected fog on fluoridation. In addition to this, the trichome structure on the ribs can produce better fluoridation in the effect of UV irradiation, which improves its durability. By comparing the contact angles of samples U, V, and W, it was found that the combination of low surface energy and rough microstructure can significantly improve the hydrophobicity. By comparing the samples with the Sarracenia bionic structure, it was found that the maximum contact angles were 163° and 160.5° before and after the fog-collection test, respectively, and the fog collection had a certain effect on it. The hydrophobicity of Class B samples was better before fog collection, and the hydrophobicity of the samples after fog collection was basically the same, demonstrating that the rib height ratio and the low number of low ribs did not have much effect on the hydrophobicity. In the adhesion test experiments, except for samples U and V, all the samples before and after the fog collection showed less degree of water droplet deformation and no droplet remained on the surface, indicating that the samples have low adhesion, as shown in Figure 5b. Figure 5c shows the rolling of the droplets of the samples on the inclined surface, with the rolling angles ranging from 8.4° to 17.2° after fog collection and as low as 6.5° before fog collection.

**Figure 6:**
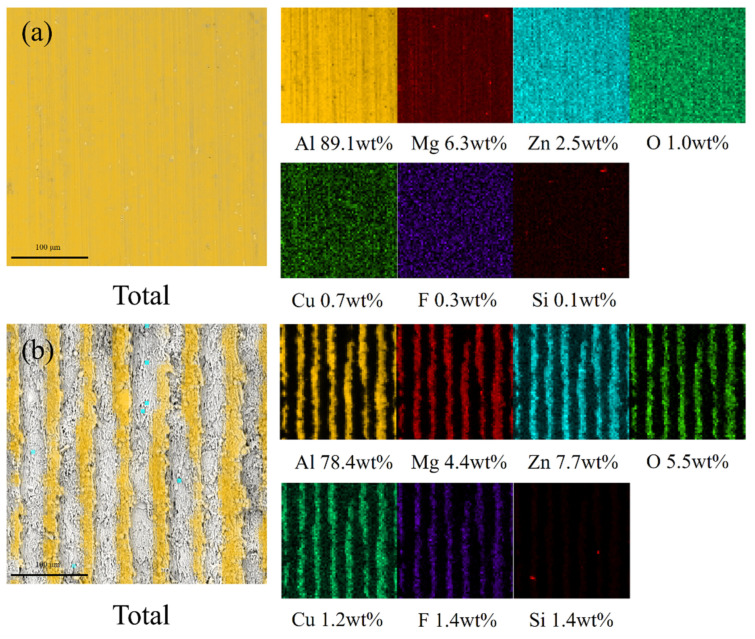
SEM images of sample and EDS elemental analysis of Al, Mg, Zn, O, Cu, F, Si. (**a**) Sample V. (**b**) Sample W.

Figure 6 shows the EDS elemental analysis of samples V and W after fog collection, which shows that the oxygen content increases from 1% to 5.5% after laser ablation, indicating that the ribs on the surface of sample W oxidize to form a trichome structure. The fluorine content is 0.3% and 1.4%, respectively, indicating that sample W is less damaged by fog collection than sample V and has better durability. This suggests that the surfaces prepared by the combination of low surface energy and rough microstructure still have good superhydrophobicity after fog collection.

### 3.3. Fog Collection Behaviour

**Figure 7:**
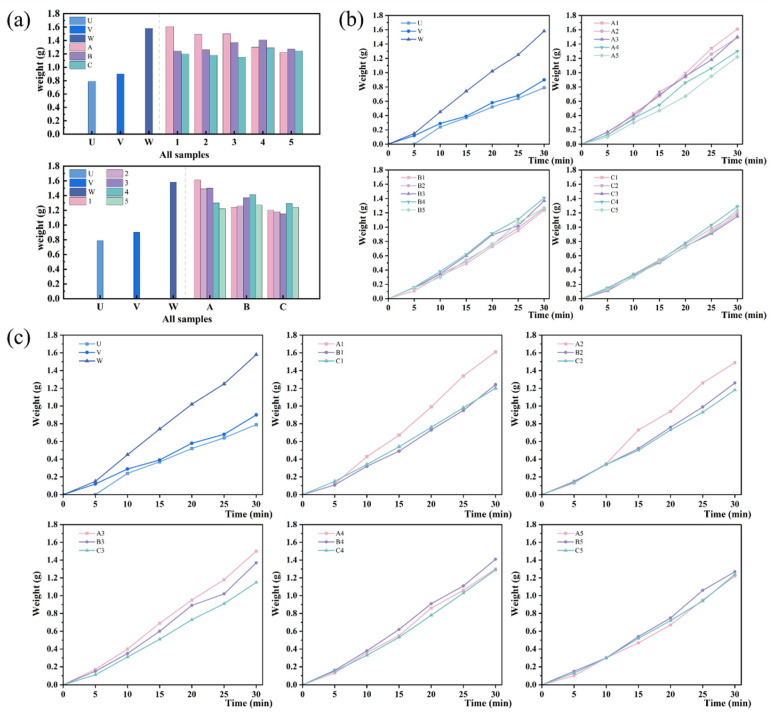
Weights of fog collection and change curves of different samples in 30 min. (**a**) Graphs of weights of fog collection at 30 min. (**b**,**c**) Graphs of change curves of fog collection in 30 min.

Figure 7 shows the graphs comparing the weight of fog collection and changes in the weight of fog collection for different samples over 30 min from both low rib number and rib height ratio, with the gradual increase of water collected on the surface over time, and there is a significant difference in the weight of water collected on different surfaces. Among them, Figure 7a shows the results at 30 min of fog collection, and Figure 7b,c show the change curves of fog collection 30 min obtained after recording the fog collection at all time points with a time interval of 5 min. From Figure 7a, it can be seen that the changes in the fog collection of the control samples U, V, and W coincided with the changes in their contact angle sizes, with the fog collection of 0.79 g, 0.9 g, and 1.58 g, and the fog-collection efficiencies of 395 mg cm^−2^ h^−1^, 450 mg cm^−2^ h^−1^, and 790 mg cm^−2^ h^−1^, respectively, which indicated that the surface wettability changed from hydrophilic to hydrophobic to super-hydrophobic, with a significant effect on the fog collection. Hydrophobicity has a significant effect on fog collection. However, when observing the fog collection of the samples with different rib height ratios, it is found that the changes in the fog collection is not completely consistent with the changes in the contact angle sizes after the surface reaches superhydrophobicity, which indicates that the surface microstructure is the main influencing factor of the fog collection at this time. Samples A1, A2, and A3 have the highest fog collection among samples 1, 2, and 3, while samples B4 and B5 have the highest fog collection among samples 4 and 5. In addition to this, as the rib height ratio decreases, the change in fog collection varies with the number of low ribs. Samples A continue to decrease, samples B increase and then decrease, and samples C show a jump. The above-observed phenomena differed from the phenomena studied by Chen et al. [17]. Their data showed that microchannels with the coefficient of the rib height difference, *k*, at 1.35 and 1.60 showed the best performance in droplet transport distance for samples B, whereas samples A showed the best performance when *k* was at 0.60, 0.85, and 1.10, and the flux ratio coefficients of all samples were increased as the value of *k* was increased, so that increasing rib height ratios are favorable to increase the flux. The calculated coefficient of the rib height difference *k* in the microchannel is shown in Figure 3d, and *k* is up to 1.18, but it is not the best effect for samples A. Therefore, the analysis suggests that the fog-collection behavior is affected by both the condensation of fog on the surface of the sample and the directional transport of the droplets off the surface of the sample. Samples C show a jump change since with the increase of the number of the low ribs, the bionic Sarracenia structure has the ultrafast transport mode in which the water film is not stabilized, resulting in a decrease in the effect of droplet directional transport. In contrast, the fog collection of samples A continued to decrease with decreasing rib height ratio under the stabilized water film, which is consistent with the phenomenon obtained by Chen et al. and indicates that the flux plays a dominant role at this time. It is worth noting that, for all the samples with Sarracenia bionic structure, only sample A1 has a higher fog collection than sample W, with a fog-collection efficiency of 805 mg cm^−2^ h^−1^, an improvement of only 1.8%, while sample C3 has the lowest fog collection, with a fog-collection efficiency of 575 mg cm^−2^ h^−1^, a reduction of 27.3%. It shows that the *k* of the prepared sample is at least 1.18 and above to reflect the ultrafast water transport in the Sarracenia bionic structure, which is characterized by the unique water film mode, conversely, because the appearance of high ribs leads to the number of channels becoming less, which reduces the flux from the influence of the fog-collection effect. Therefore, the further increase of the rib height ratio is an effective means to improve the fog-collection efficiency. From Figure 7b,c, it can be seen that sample U has no droplets slipping off the surface at 5 min of fog collection due to surface hydrophilicity and high adhesion, and the slopes of the change curves of the fog-collection weights of all the samples are more uniform, which indicates that the process of the establishment of the water film is faster. Figure 7b shows that the change curves of fog collection with different low rib height ratios converge and the slopes decrease when the rib height ratio decreases. Figure 7c shows that the change curves of fog collection with different rib height ratios converge and the slopes decrease when the number of low ribs increases. It is analyzed that the shape of the water film is also one of the main factors affecting the fog collection, and the decrease of the rib height ratio and the increase of the low ribs number will make the water film become smooth, and the effect of ultrafast water transport decreases.

**Figure 8:**
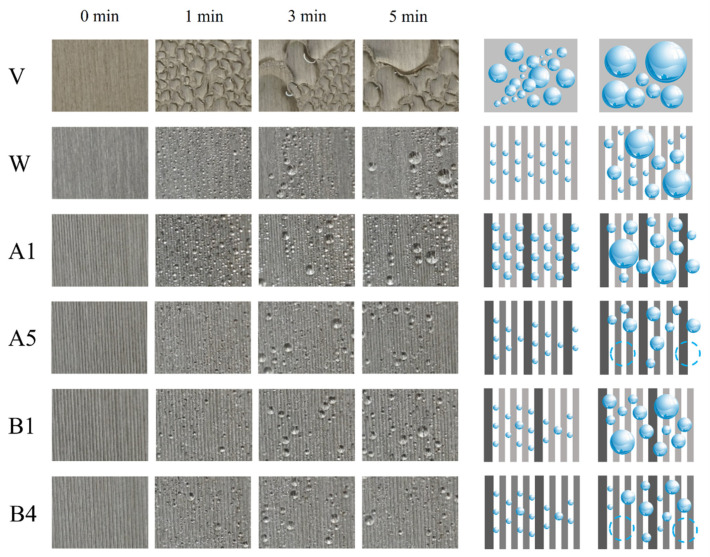
Fog-collection process for different samples.

To better investigate the effects of fog condensation on the sample surface and the directional transport of droplets away from the sample surface on fog collection, the fog-collection processes of samples V, W, A1, A5, B1, and B4 were observed for nucleation, growth, and detachment from the surface, as shown in Figure 8. When the fog contacts the samples, the fog condenses into tiny droplets that adhere to the surface (1 min). As the small droplets grow, polymerization occurs between the droplets (3 min). Finally, the droplets increase in size to a certain degree and quickly slide off and leave the surface under the effect of gravity (5 min). From the comparison of sample V with sample W in Figure 8, it can be seen that the fog tends to condense directionally at the ridge of the groove, showing homogeneity on the all low rib surface and randomness on the smooth surface, indicating that the groove facilitates the collection of fog. In addition, by comparing sample W with sample A1, it can be seen that the droplets on the high-low rib surfaces are larger than those on the all low rib surface during droplet nucleation and growth (1 min, 3 min), indicating that the hierarchical groove surface favors the condensation of droplets. This is due to the fact that when the surface of sample A1 is just in contact with the fog, because the surface has micrometer structures and the air in this structure occupies a large area, following the principle of minimum energy effect, the initial contact state of the droplets formed after the fog condenses on the surface (between high ribs, low ribs, and high-low ribs) are all in the Cassie state. Due to the ridge-like protrusion of the groove out of the surface, the ridge-like protrusion concentrates the fog flow above it. The high-low ribs structure on the surface of the hierarchical groove can capture more fog flow, and the gravity of the droplets increases after absorbing the fog flow. The fog continues to condense so that the droplets penetrate the air to form the Cassie–Wenzel state, then the contact state of droplets on the surface is a mixture of the Cassie–Wenzel state and the Wenzel state. Finally, under the continuous penetration of fog, all the droplet contact states are completely transformed into the Wenzel state, and the hierarchical structure of high-low ribs, and the interception effect of the high ribs on the directional coagulation of droplets, make it easier for the small droplets to be caught by the ridges and accelerate the growth of droplets so that droplets will have a larger volume in coagulation. Comparison of the other samples except sample A1 with sample W shows that the droplet volume of sample W is larger in all phases of the fog-collection process, which is due to the lower height of the ribs. The droplets condense faster, and their contact state is easier to reach the Wenzel state, so the fog-collection effect is better, which also corresponds to the data of the fog collection in Figure 7.

A comparison of sample A1 and sample B1 shows that the droplets condensed are smaller in size in the fog on the surface of sample B1, but the number of large droplets is greater. This may be due to the increase in the number of low ribs, because the microstructure area is the same, the number of high ribs decreases, and the whole surface presents a decrease in the intercepting capacity of the high ribs, resulting in a decrease in the droplet anisotropic condensation effect and the droplet condensation of the droplet size becomes smaller. At the same time, the number of low ribs increases, the distance and space between the high ribs become larger, and the three-phase line of the droplet arrives at the high ribs and stagnates for a longer period, which leads to a more significant pinning phenomenon on the surface of the droplet and the formation of more large droplets. Comparison of sample A1 with sample A5 and sample B1 with sample B4 shows that sample A5 and sample B4 are significantly affected by the phenomenon of droplets detaching from the surface by gravity, which may be attributed to the reduction of the rib height ratio. The space between the high ribs becomes smaller, and the three-phase lines of droplets arrive at the high ribs with less time for the process of stagnation, which results in the alleviation of the phenomenon that droplets are pinning on the surface, so droplets are more likely to detach from the surface of the sample, which is favorable to the formation of the water film. However, the above data of fog collection showed a decreasing trend from sample B1 to sample B4, and it was analyzed that the flux is the main influencing factor, while increasing the coefficient of the rib height difference *k* and rib height ratio in the microchannels can increase the flux, and at this time, the shape of the water film will be changed, so the formation, shape, and stability of the water film is an important factor influencing the surface fog collection of the bionic Sarracenia structure.

**Figure 9:**
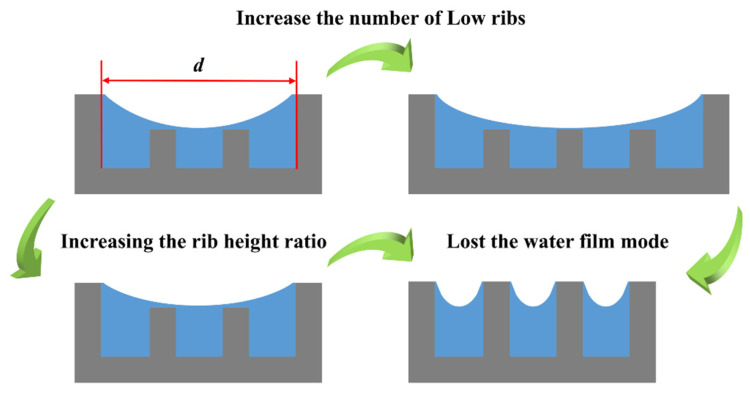
Effect of water film shape on fog collection.

To better study the effect of water film on fog collection, the water film width *d* is defined, as shown in Figure 9. The water film width *d* can be expressed by Equation (1):

(1) $$d=nw_{r}+\left(n+1\right)w$$

where *n* is the number of low ribs and $w_{r}$ is the width of the low ribs.

The aspect ratio *D* for the shape of the water film can be further derived and can be expressed by Equation (2):

(2) $$D=\frac{H-h}{d}=\frac{H-h}{nw_{r}+\left(n+1\right)w}$$

A comparison of the above sample W and sample A1 shows that the bionic Sarracenia structure of sample A1 can reflect the effect of the water film mode in the fog collection, so it can be used as the lowest value of the effective water film, and the value of *D* is 0.144. When the value of *D* is greater than 0.144, the formation of water film is faster and more stable, which is conducive to the directional transport of droplets, and the effect of the hierarchical structure of the fog collection is good. When *D* is less than 0.144, the water film mode cannot play its ultrafast transport feature in the fog collection, and the effect of the hierarchical structure of the fog collection is poor. When the *D* value is too low, the water film mode is gradually lost, and the fog-collection effect of hierarchical structures is no different from single structures.

## 4. Conclusions

In summary, in this study, the surfaces of the bionic Sarracenia structure for fog collection were prepared by laser ablation on the surface of aluminum alloys. After UV irradiation and fluoridation, the prepared surfaces have superhydrophobicity and low adhesion with an average sliding angle of 10°. The effect of the bionic Sarracenia structure on fog collection was explored from two perspectives, i.e., low ribs number and rib height ratio, and it was found that the fog-collection behavior was mainly caused by a combination of droplet directional condensation and droplet directional transport, of which the latter was not only controlled by the flux, but also correlated with the shape of the water film. To investigate how to better realize the ultrafast directional transport of droplets in the water film mode with the hierarchical structure of bionic Sarracenia, the aspect ratio *D* of the water film is defined and its relationship with the fog-collection effect is given. In addition, a cyclic fog-collection test was conducted to verify its fog-collection capability over a long period. The results show that after 15 cycle tests, the fog-collection efficiency of sample A1 is still 750 mg cm^−2^ h^−1^, which is only an 8.1% loss compared to the first time, thus revealing the durability of the sample surface. This work makes the surface functionalization of various metal materials possible, which provides better references and thoughts for researchers aiming to prepare fog-collection surfaces, especially in the fields of biomedical and microfluidic devices.

## Data Availability

Data will be made available on request.
